# Crystal structure of ATP-dependent DNA ligase from *Rhizobium* phage vB_RleM_P10VF

**DOI:** 10.1107/S2053230X2500411X

**Published:** 2025-05-14

**Authors:** Ulli Rothweiler, Hanna-Kirsti S. Leiros, Adele Williamson

**Affiliations:** a ArcticZymes Technologies ASA, 9037Tromsø, Norway; b The Norwegian Structural Biology Centre UiT The Arctic University of Norway Forskningsparken 3 9037Tromsø Norway; c School of Science University of Waikato Hamilton3216 New Zealand; Sungkyunkwan University School of Medicine, Republic of Korea

**Keywords:** *Rhizobium* phage, DNA ligases

## Abstract

DNA ligases are foundational molecular-biological tools used for cloning and sequencing workflows, and are essential replicative enzymes for all cellular life forms as well as many viruses and bacteriophage. There is considerable interest in structurally and functionally characterizing novel DNA ligases and profiling their suitability for molecular-biological applications. Here, we report the crystal structure of the ATP-dependent DNA ligase from the *Rhizobium* phage vB_RleM_P10VF bound to a nicked DNA duplex determined to 2.2 Å resolution. The enzyme crystallized in the DNA-encircling conformation, arrested as a step 2 intermediate in the catalytic cycle with the adenylating cofactor transferred to the 5′-phosphate of the DNA nick. The overall structure of the DNA ligase closely resembles that of the T4 DNA ligase, including an α-helical globular DNA-binding domain. Several secondary-structural elements are abbreviated in the P10VF DNA ligase relative to the T4 DNA ligase enzyme, which may account for its lower specific activity, especially on DNA substrates containing double-stranded breaks.

## Abstract 2

We have determined the structure of the *Rhizobium* phage vB_RleM_P10VF DNA ligase bound to a nicked DNA duplex to 2.2 Å resolution. The DNA ligase exhibits a canonical DNA ligase-binding mode fully encircling the duplex and has considerable structural homology to T4 DNA ligase and the bacterial ATP-dependent DNA ligase from *Prochlorococcus marinus*.

## 1. Introduction

DNA ligases are enzymes which join breaks in the phosphodiester backbone of double-stranded DNA using an adenylate-donating cofactor: either ATP or NAD^+^ (Tomkinson *et al.*, 2006 ▸). The ATP-dependent class of DNA ligases are widespread in biology and are found in all archaea and eukaryotes as well as a large number of bacteria and viruses (Williamson *et al.*, 2016 ▸; Pergolizzi *et al.*, 2016 ▸). The DNA ligases of viruses, and in particular those from bacteriophages, have played a central role in the development of molecular-biological workflows (Lohman *et al.*, 2011 ▸). This includes the original procedure for generating recombinant DNA *in vitro* using restriction-ligation cloning (Weiss & Richardson, 1967 ▸) through to more recent innovations in DNA assembly such as the Gibson (isothermal assembly) and Golden Gate (Type IIS cloning) methodologies (Gibson *et al.*, 2009 ▸; Potapov *et al.*, 2018 ▸). The most widely used commercial DNA ligase is T4 DNA ligase, although other viral enzymes have found niche applications. These include T3 DNA ligase, which exhibits salt tolerance, T7 DNA ligase, which preferentially ligase nicks and cohesive ends, providing extra stringency, and Chlorella virus ligase, which is sold as SplintR Ligase and is used in the detection of specific RNA sequences due to its ability to ligate adjacent DNA strands which are annealed to an RNA complement (Lohman *et al.*, 2011 ▸; Bauer *et al.*, 2017 ▸). A recently described DNA ligase from *Chronobacter* phage, supplied commercially as R2D Ligase, has the unique ability to ligate an RNA strand to either end of a DNA strand when annealed to a DNA template and may find applications in sequencing technologies (Gundesø *et al.*, 2024 ▸).

T4 DNA ligase is an essential enzyme in the replicative lifecycle of the T4 bacteriophage, where it functions to join Okazaki fragments in conjunction with RNaseH (Miller *et al.*, 2003 ▸). The crystal structure of T4 DNA ligase showed that it possesses an α-helical DNA-binding (DB) domain which is appended to the N-terminus of its core catalytic nucleotide-transferase (NTase) and oligonucleotide-binding (OB) domains (Shi *et al.*, 2018 ▸). Together, these three domains form an encircling clamp around the DNA duplex which is completed by noncovalent interactions between the DB and OB domains. This mode of encirclement is broadly common to almost all structurally characterized DNA ligases, although it may be achieved by different domain architectures, including DB domains that contain β-structures, C-terminal globular domains or unstructured ‘latch’ regions which protrude form the core domains (Williamson & Leiros, 2020 ▸). The globular DB domain of T4 is the ‘archetypal’ helical DB domain of DNA ligases, which is also found in the archaeal replicative DNA ligases and larger eukaryotic ligases, including the three human forms (Shi *et al.*, 2018 ▸).

Given the widespread use of bacteriophage DNA ligases in molecular biology, there is considerable interest in exploring the diversity of structure and function among additional bacteriophage DNA ligases. Here, we present the crystal structure of the ATP-dependent DNA ligase from *Rhizobium* phage vB_RleM_P10VF (P10VF-Lig), which has substantial structural similarity to T4 DNA ligase as well as to the ATP-dependent DNA ligase from the cyanobacterium *Prochloro­coccus marinus* (Pmar-LigP), despite having low overall sequence identity to either protein.

## 2. Materials and methods

### 2.1. Macromolecule production

The gene encoding the DNA ligase P10VF-Lig from *Rhizobium* phage vB_RleM_P10VF (YP_009099956) was synthesized by GeneArt (Life Technologies) with codon optimization for expression in *Escherichia coli*. The clonal gene, which also encoded an N-terminal cleavage site for the Tobacco etch virus (TEV) protease, was purchased pre-cloned into pDONR221 entry vectors and then subcloned into the pDEST17 expression vector using Gateway recombination.

Production of selenomethionine (SeMet)-substituted P10VF-Lig for crystallization was performed by cultivation of *E. coli* BL21(DE3) pLysS cells in M9 minimal medium at 37°C until an OD_600_ of 0.4 was reached. After this time, supplementary amino acids were added at the following concentrations: l-lysine, l-phenylalanine and l-threonine at 100 mg ml^−1^, isoleucine, l-leucine and l-valine at 50 mg ml^−1^ and l-selenomethinone at 60 mg ml^−1^. Cultivation was continued for 1 h at 37°C; the temperature was then decreased to 15°C and expression was induced by the addition of 0.1 m*M* isopropyl β-d-thiogalactopyranoside and allowed to proceed overnight. SeMet-substituted P10VF-Lig was purified and detagged as described for the native protein (Section S1). Pooled protein-containing fractions after gel filtration were concentrated to 200 µ*M* and flash-frozen in liquid nitrogen before storage at −80°C.

Synthetic DNA oligonucleotides were ordered from Integrated DNA Technologies (IDT) with HPLC purification. A 2′-*O*-methylation (2′-*O*-Me) modification was included one nucleotide upstream from the 3′-terminus of the nick as this substrate has been successfully used to co-crystallize other DNA ligase–DNA substrate complexes (Nair *et al.*, 2007 ▸; Nandakumar *et al.*, 2007 ▸). Macromolecule-production information is summarized in Table 1 ▸.

### 2.2. Crystallization

To generate the nicked DNA duplex, single oligonucleotides were resuspended at 9 m*M* in annealing buffer (50 m*M* Tris pH 8.0, 50 m*M* NaCl, 1 m*M* EDTA), mixed in a 1:1:1 ratio to give a final duplex concentration of 3 m*M* and incubated at 85°C before cooling overnight. P10VF-Lig was incubated with 1.2 molar equivalents of nicked duplex and 5 m*M* additional EDTA for 30 min on ice to form the protein–DNA complex. Crystals were grown by the hanging-drop diffusion method at 4°C from 24% PEG 4000, 100 m*M* bis-Tris pH 5.5 and appeared within a few days. Crystals were cryoprotected in mother liquor with an additional 12% ethylene glycol and were flash-cooled in liquid nitrogen. Crystallization information is summarized in Table 2 ▸.

### 2.3. Data collection and processing

Diffraction data to 2.2 Å resolution were measured on beamline 14.1 at BESSY II, Berlin. The crystal was cooled to 100 K and data were recorded on a Dectris PILATUS3 6M detector using X-rays at a wavelength of 0.979839 Å. Data were integrated, scaled and truncated in *XDSapp* (Kabsch, 2010 ▸) and merged using *AIMLESS* (Evans & Murshudov, 2013 ▸). Data-collection and processing statistics are summarized in Table 3 ▸.

### 2.4. Structure solution and refinement

The complex structure of SeMet P10VF-Lig was solved by single-wavelength anomalous diffraction (SAD) using *phenix.autosol* and further refined with *phenix.refine* (Afonine *et al.*, 2012 ▸; Terwilliger *et al.*, 2009 ▸) with iterative rounds of model building in *Coot* (Emsley *et al.*, 2010 ▸). The *I*/σ(*I*) in the outer shell is 1.84. It falls below 2 in the second outermost shell at 2.33 Å resolution. The *I*/σ(*I*) in the second outermost shell is 3.22. The resolution cutoff was chosen by *XDSapp* (Kabsch, 2010 ▸). Refinement statistics are summarized in Table 4 ▸.

## 3. Results and discussion

**Figure 1:**
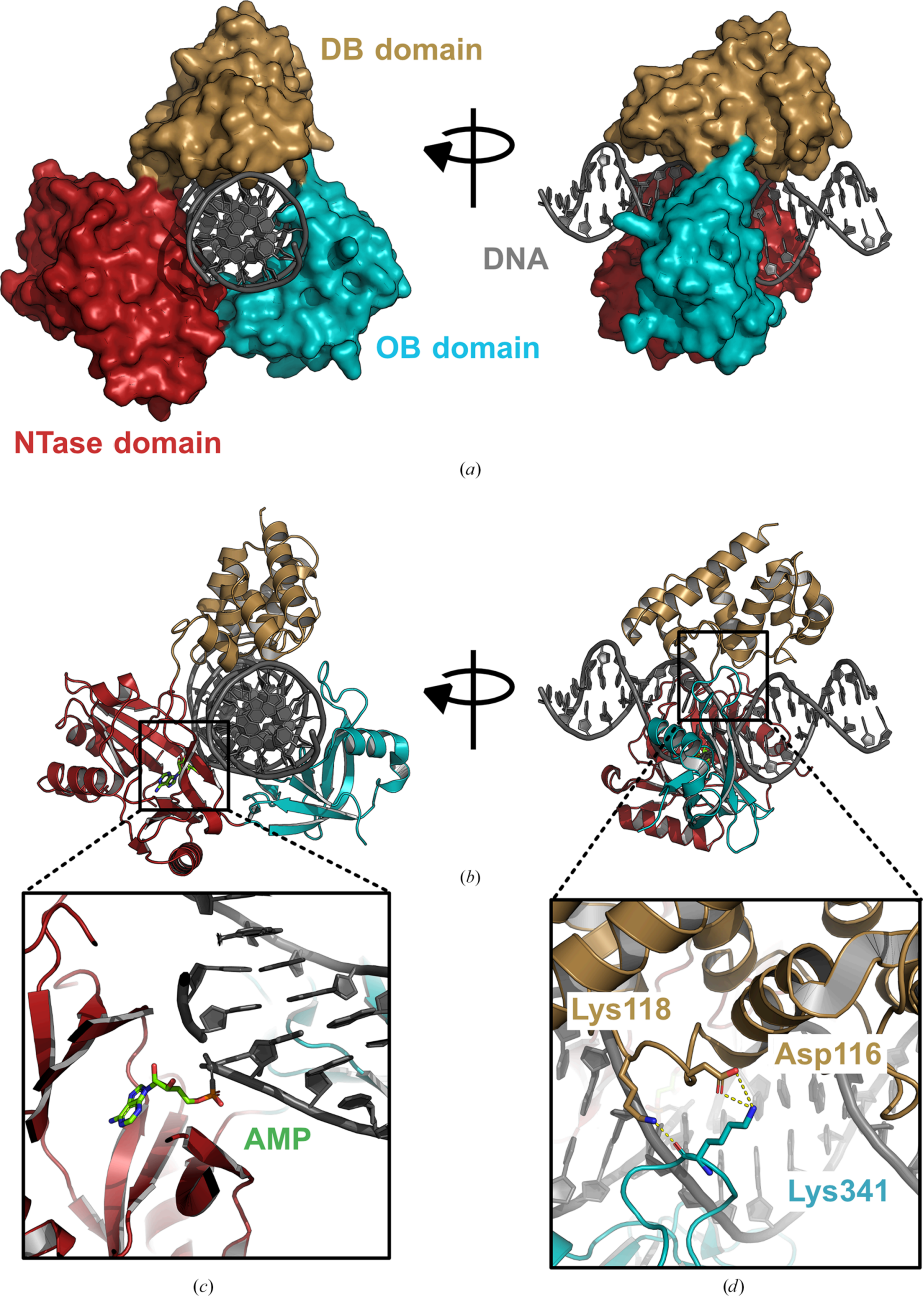
Overall structure of P10VF-Lig bound to nicked DNA. (*a*) DNA ligase shown as a surface. (*b*) DNA ligase rendered as a cartoon to show secondary-structure elements. (*c*) Detailed view of the 5′ DNA–adenylate ‘step 2’ intermediate captured in the crystal structure. (*d*) Detailed view of noncovalent interactions between the DB and OB domains which complete the encirclement of the DNA duplex by P10VF-Lig. In all figures the DNA-binding domain is coloured gold, the nucleotidyl transferase domain is coloured red and the oligonucleotide-binding domain is coloured teal. DNA is shown in grey and AMP in green.

The P10VF-Lig DNA ligase was crystallized in the closed conformation bound to nicked DNA. Analysis of the overall structure revealed that the DNA duplex was encircled by the three globular domains: the highly conserved NTase domain, C-terminal OB domain and N-terminal DB domain (Fig. 1 ▸*a*). As anticipated, the latter domain comprises eight α-helices, with the majority of the protein–DNA contacts from this domain being formed by inter-helical loops (Fig. 1 ▸*b*).

The complex has crystallized as the ‘step 2’ intermediate of the DNA ligase reaction where the AMP moiety has been transferred from the active site of the ligase enzyme to the 5′-phosphate of the DNA substrate (Fig. 1 ▸*c*). This intermediate has been crystallized in a number of other DNA ligase–DNA substrate co-crystal structures (Shi *et al.*, 2018 ▸; Williamson & Leiros, 2019 ▸; Williamson *et al.*, 2018 ▸) and is considered to result from covalent adenylation (step 1 reaction) of the DNA ligase enzyme during protein purification prior to its addition to the crystallization experiment. The encirclement of the nicked DNA duplex is completed by noncovalent interactions between side chains of the DB domain (Asp116 and Lys118) and OB domain (Lys341) (Fig. 1 ▸*d*).

**Figure 2:**
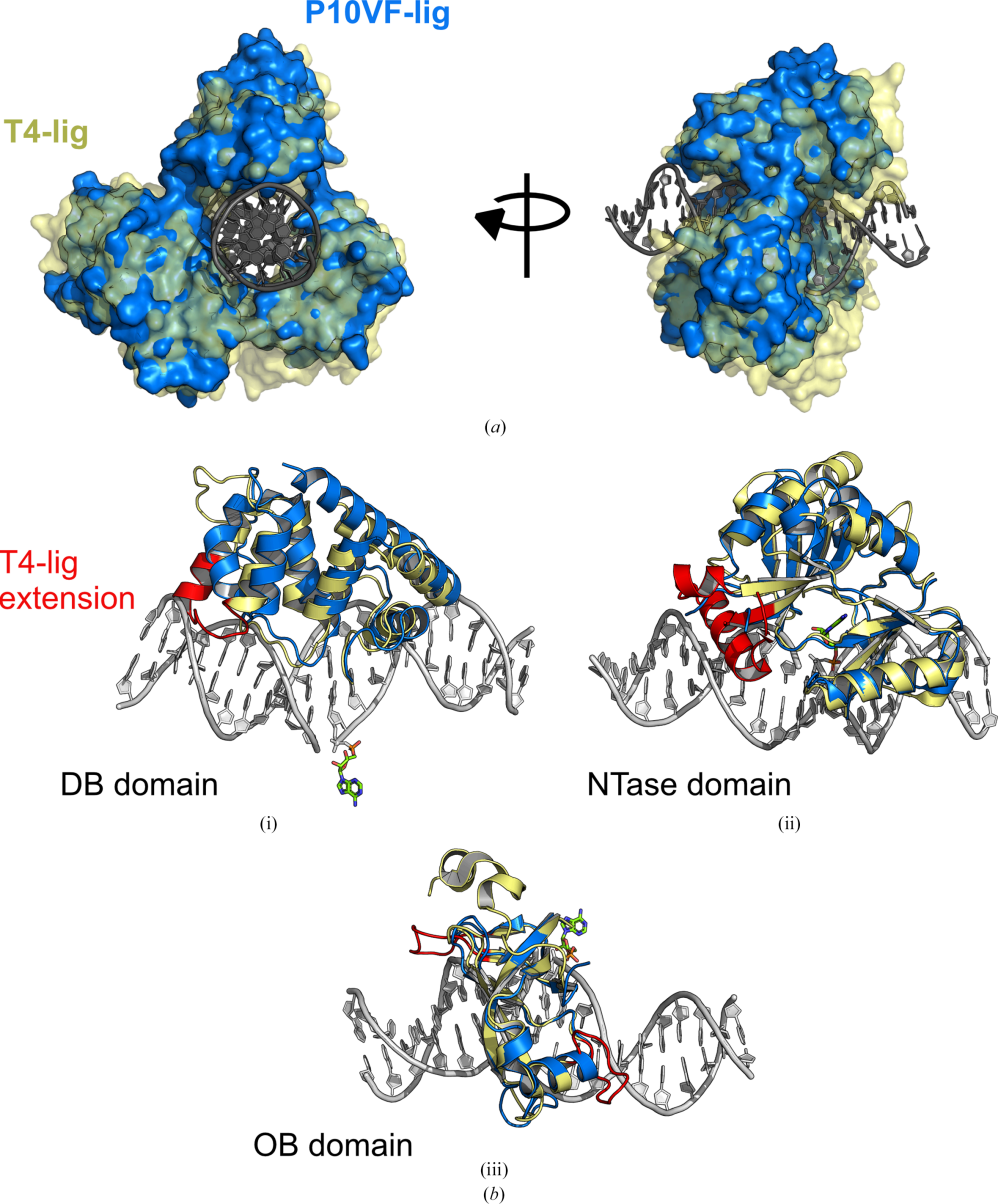
(*a*) Structural overlay of P10VF-Lig (blue) with T4 DNA ligase (yellow), highlighting the relatively more extensive surface of the T4 enzyme. DNA in this view (grey) is shown for the P10VF-Lig structure only, but is essentially identical to the T4 DNA ligase structure when superimposed. (*b*) Structural overlay of P10VF-Lig with T4 DNA ligase for individual domains rendered in cartoon mode to highlight secondary-structural elements. Features which are present in T4 DNA ligase but absent in P10VF-Lig are coloured red. DNA adenylate is shown for the P10VF-Lig structure only.

Structural alignment of P10VF-Lig with T4 DNA ligase revels that the latter has a more extensive binding surface with the DNA substrate (Fig. 2 ▸). This is reflected in the relative buried solvent-accessible surface areas of the DNA substrate by each protein, with only 901 Å of the DNA substrate (18.3%) interfacing with the P10VF-Lig protein, compared with 1406 Å (29.2% of the DNA duplex) buried by the T4-DNA ligase protein (Supplementary Table S2). This discrepancy is due to differences in helix 4 of the DB domain, which is truncated and kinked in P10VF-Lig relative to the equivalent secondary structure in T4 DNA ligase (Fig. 2 ▸*b*, i), as well as two loops in the OB domain of T4 DNA ligase (Fig. 2 ▸*b*, iii). The NTase domain of T4 DNA ligase also contains additional helical elements relative to P10VF-Lig which insert into the minor groove on the 3′-end of the DNA nick. In P10VF-Lig the equivalent 17 residues between Ala212 and Asn229 have no visible density (Fig. 2 ▸*b*, ii). In T4 DNA ligase, this region also contains an unstructured gp45 clamp-binding motif which has previously been shown to facilitate interaction between the clamp and the ligase, potentially increasing the processivity of the enzyme; however, there is no equivalent clamp-binding motif in the sequence of P10VF-Lig (Supplementary Fig. S1).

**Figure 3:**
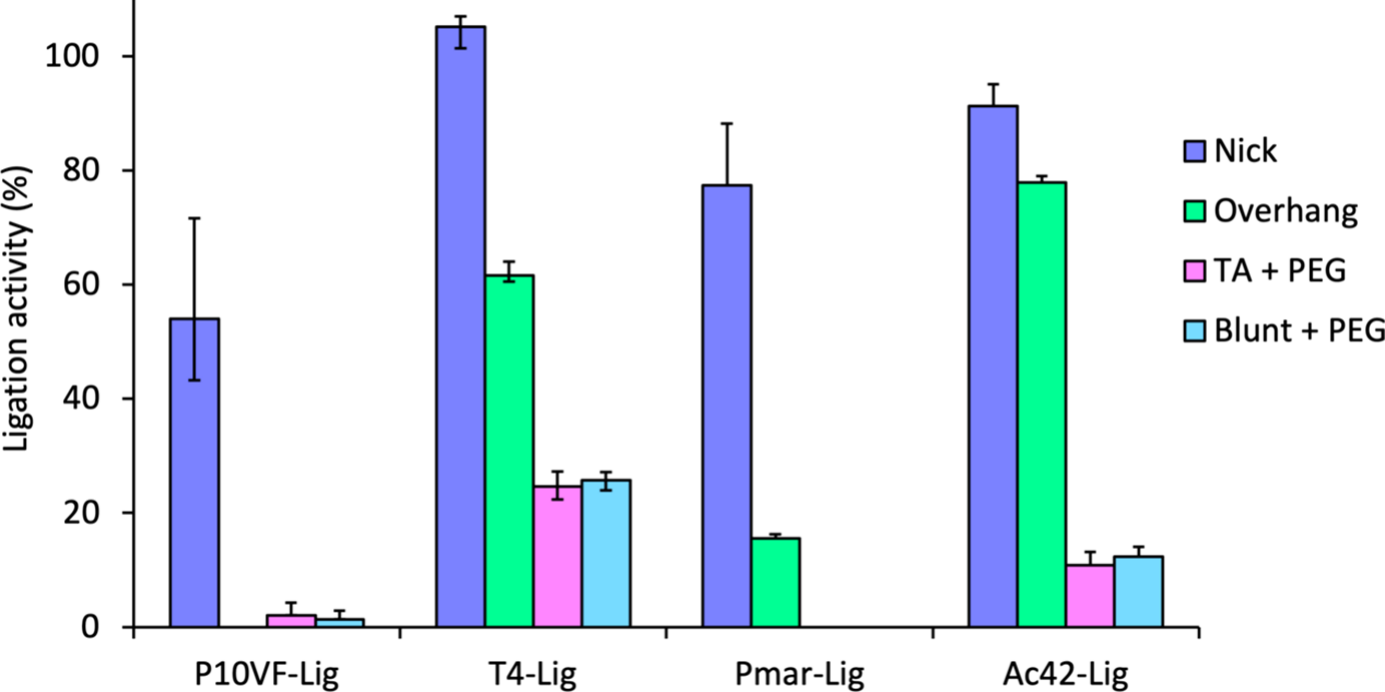
DNA-ligation activity of different DNA breaks by T4-like DNA ligase enzymes. The Nick substrate contained a break on a single strand of the DNA duplex. The overhang substrate contained a cohesive double-stranded break with a four base-pair overhang, the TA + PEG substrate contained a cohesive double-stranded break with a single T–A overhang and included 10%(*w*/*v*) PEG 3350, and the Blunt + PEG substrate contained a double-stranded break without any cohesive overhang and included 10%(*w*/*v*) PEG 3350. Details of the assay conditions are given in Section S2. Values represent the mean of duplicate measurements and error bars represent the standard deviation from the mean.

P10VF-Lig DNA ligase was originally selected for *in vitro* characterization on the basis that it aligns with T4 DNA ligase and other T4-like DNA ligases for the entirety of its amino-acid sequence, albeit with a residue identity of less than 18% (Supplementary Table S1). Given the widespread use of bacteriophage enzymes in molecular biology, we aimed to determine whether T4 DNA ligase homologs such as P10VF-Lig DNA ligase might possess similarly high activities and broad substrate ranges to the T4 DNA ligase enzyme (Bullard & Bowater, 2006 ▸). To this end, P10VF-Lig and a second novel phage-derived T4-DNA ligase homolog from *Acinetobacter* phage Ac42 (Ac42-Lig) were assayed on a range of ligatable DNA substrates and compared with T4 DNA ligase and the bacterial ATP-dependent DNA ligase Pmar-LigP. Despite the relatively high structural similarity between P10VF-Lig and T4 DNA ligase, P10VF-Lig exhibited considerably lower specific activity on most substrates tested, with no detectable ligation on cohesive overhangs (Fig. 3 ▸). Ac42-Lig, however, exhibited robust activity on all four substrates that was comparable to that of T4 DNA ligase. This may be due to the different relative binding areas of these respective DNA ligases with the DNA substrate; Ac42-Lig has equivalent insertions in its NTase and OB domains as T4 DNA ligase, while both P10VF-Lig and Pmar-LigP have similarly abbreviated structures in these regions and both exhibit low activity on double-stranded breaks (Fig. 3 ▸; Supplementary Fig. S1).

In conclusion, our structural and functional analysis of these T4 DNA-ligase homologs indicates that the differing abilities of DNA ligases to join DNA substrates may be due to relatively subtle changes in their binding surfaces. This validates the analysis of DNA ligase–DNA substrate complexes as part of a comprehensive strategy towards the discovery or design of better DNA ligase enzymes. Such insight may prove especially powerful in the context of ever-improving structural modelling by allowing high-throughput models to be benchmarked against validated crystal structures of biochemically characterized DNA ligase enzymes. Such comprehensive information will be useful to inform sequence-based bio-discovery endeavours, as well as structure-guided design strategies.

## 4. Related literature

The following references are cited in the supporting information for this article: Krissinel & Henrick (2007 ▸), Robert & Gouet (2014 ▸) and Stelzer *et al.* (2024 ▸).

## Supplementary Material

PDB reference: *Rhizobium* phage ligase, 9fzx

Supplementary Sections S1 and S2, Supplementary FIgure S1 and Supplementary Tables S1 and S2. DOI: 10.1107/S2053230X2500411X/ek5039sup1.pdf

## Figures and Tables

**Table 1:** Macromolecule-production information **M** indicates selenomethionine substitution; (P) indicates 5′-phosphorylation of the subsequent nucleotide; (2-Ome) indicates 2′-*O*-methylation of the preceding nucleotide.

Source organism	*Rhizobium* phage vB_RleM_P10VF
DNA source	Synthetic
Cloning vector	pDONR221
Expression vector	pDEST17
Expression host	*E. coli* BL21(DE3) pLysS
Complete amino-acid sequence of the construct produced	GLDIFSDNVSEINRISDIINSDLQAIADSKGTNAKKVELAKISEYTFKCFVFHLDPFQNFGISKLSKDAGGGEGIDWSTVFKLLYEGKGRDLKKRDKSLTTLQAKIINGIFDGF**M**DWKPGVKGGSFLDVFPDSYRTFEVQKCANWDPDLFEANSFAQIKFDGIRCVA**M**VDHNGNLTYVSRNGKPVVNIDPRIEEN**M**KLHPGWCFDAEADSPAKFQKTSGISRASKSGSNIKLTLRVFDAIPYDAFLARKYDVQYIERYNDLKS**M**WSNNPFLFDLIADHTLVETWEDAQKFYEDSRANGNEGAIVKKRFGTYNFGRDDSW**M**KVKPLETIEARIIGYEEGKPKTKHVGRVGALIVQDYTGAISRVGSG**M**SDKERQYIYDNWDEFENALCEVKF**M**ERTESGVFRHSRLSKIRLDKDD**M**NPTGA
DNA sequence of oligonucleotide	Complement: TTCCGACAGTGGGGTCGCAAT
	3′ of nick: ATTGCGACC(2-Ome)C
	5′ of nick: (P)CACTATCGGAA

**Table 2:** Crystallization

Method	Vapour diffusion, hanging drop
Plate type	24-well VDXm plate
Temperature (K)	278
Protein concentration (mg ml^−1^)	10
Buffer composition of protein solution	50 m*M* Tris pH 7.5, 200 m*M* NaCl, 1 m*M* β-mercaptoethanol
Composition of reservoir solution	24% PEG 4000, 100 m*M* bis-Tris pH 5.5
Volume and ratio of drop	2 µl + 2 µl
Volume of reservoir (µl)	500

**Table 3:** Data collection and processing

Diffraction source	BESSY beamline 14.1
Wavelength (Å)	0.979839
Temperature (K)	100
Detector	Dectris PILATUS3 6M
Crystal-to-detector distance (mm)	458.87
Rotation range per image (°)	0.1
Total rotation range (°)	720
Exposure time per image (s)	0.08
Space group	*P*2_1_2_1_2_1_
*a*, *b*, *c* (Å)	68.04, 100.39, 107.69
α, β, γ (°)	90, 90, 90
Mosaicity (°)	0.078
Resolution range (Å)	50–2.2 (2.3–2.2)
Total No. of reflections	989031
No. of unique reflections	72435
Completeness (%)	99.7 (98.3)
Multiplicity	13
〈*I*/σ(*I*)〉	19.56†
*R*_meas_ (%)	9.5
Overall *B* factor from Wilson plot (Å^2^)	54.13

† The *I*/σ(*I*) in the outer shell is 1.84. It falls below 2 in the second outermost shell at 2.33 Å resolution. The *I*/σ(*I*) in the second outermost shell is 3.22. The resolution cutoff was chosen by *XDSapp* (Kabsch, 2010 ▸).

**Table 4:** Structure refinement

Resolution range (Å)	47.5–2.2 (2.3–2.2)
Completeness (%)	99.6
σ Cutoff	*F* > 1.34σ(*F*)
No. of reflections, working set	72380 (4496)
No. of reflections, test set	2089 (134)
Final *R*_cryst_	0.205 (0.3709)
Final *R*_free_	0.246 (0.3869)
No. of non-H atoms	
Protein	3114
Nucleic acid	857
Ligand	36
Water	184
Total	4191
R.m.s.d., bond lengths (Å)	0.003
R.m.s.d., angles (°)	0.548
Average *B* factors (Å^2^)	
Protein	53.2
Nucleic acid	63.6
Ligand	50.5
Water	56.2
Ramachandran plot	
Most favoured (%)	97
Allowed (%)	3

## References

[bb1] Afonine, P. V., Grosse-Kunstleve, R. W., Echols, N., Headd, J. J., Moriarty, N. W., Mustyakimov, M., Terwilliger, T. C., Urzhumtsev, A., Zwart, P. H. & Adams, P. D. (2012). *Acta Cryst.* D**68**, 352–367. 10.1107/S0907444912001308 PMC3322595 22505256

[bb2] Bauer, R. J., Zhelkovsky, A., Bilotti, K., Crowell, L. E., Evans, T. C. Jr, McReynolds, L. A. & Lohman, G. J. S. (2017). *PLoS One*, **12**, e0190062. 10.1371/journal.pone.0190062 PMC5746248 29284038

[bb3] Bullard, D. R. & Bowater, R. P. (2006). *Biochem. J.* **398**, 135–144. 10.1042/BJ20060313 PMC1525015 16671895

[bb4] Emsley, P., Lohkamp, B., Scott, W. G. & Cowtan, K. (2010). *Acta Cryst.* D**66**, 486–501. 10.1107/S0907444910007493 PMC2852313 20383002

[bb5] Evans, P. R. & Murshudov, G. N. (2013). *Acta Cryst.* D**69**, 1204–1214. 10.1107/S0907444913000061 PMC3689523 23793146

[bb6] Gibson, D. G., Young, L., Chuang, R. Y., Venter, J. C., Hutchison, C. A. & Smith, H. O. (2009). *Nat. Methods*, **6**, 343–345. 10.1038/nmeth.1318 19363495

[bb7] Gundesø, S. E., Rothweiler, U., Heimland, E., Piotrowski, Y., Rødum, I. K., Söderberg, J. J., Gábor, I. M., Solstad, T., Williamson, A., Lanes, O. & Striberny, B. K. (2024). *Biotechnol. J.* **19**, 2300711. 10.1002/biot.202300711 38528369

[bb8] Kabsch, W. (2010). *Acta Cryst.* D**66**, 125–132. 10.1107/S0907444909047337 PMC2815665 20124692

[bb99] Krissinel, E. & Henrick, K. (2007). *J. Mol. Biol.* **372**, 774–797. 10.1016/j.jmb.2007.05.022 17681537

[bb9] Lohman, G. J. S., Tabor, S. & Nichols, N. M. (2011). *Curr. Protoc. Mol. Biol.* **94**, 3.14.1–3.14.7. 10.1002/0471142727.mb0314s94 21472697

[bb10] Miller, E. S., Kutter, E., Mosig, G., Arisaka, F., Kunisawa, T. & Rüger, W. (2003). *Microbiol. Mol. Biol. Rev.* **67**, 86–156. 10.1128/MMBR.67.1.86-156.2003 PMC150520 12626685

[bb11] Nair, P. A., Nandakumar, J., Smith, P., Odell, M., Lima, C. D. & Shuman, S. (2007). *Nat. Struct. Mol. Biol.* **14**, 770–778. 10.1038/nsmb1266 17618295

[bb12] Nandakumar, J., Nair, P. A. & Shuman, S. (2007). *Mol. Cell*, **26**, 257–271. 10.1016/j.molcel.2007.02.026 17466627

[bb13] Pergolizzi, G., Wagner, G. K. & Bowater, R. P. (2016). *Biosci. Rep.* **36**, 00391. 10.1042/BSR20160003 PMC5052709 27582505

[bb14] Potapov, V., Ong, J. L., Kucera, R. B., Langhorst, B. W., Bilotti, K., Pryor, J. M., Cantor, E. J., Canton, B., Knight, T. F., Evans, T. C. Jr & Lohman, G. J. S. (2018). *ACS Synth. Biol.* **7**, 2665–2674. 10.1021/acssynbio.8b00333 30335370

[bb98] Robert, X. & Gouet, P. (2014). *Nucleic Acids Res.* **42**, W320–W324. 10.1093/nar/gku316 PMC4086106 24753421

[bb15] Shi, K., Bohl, T. E., Park, J., Zasada, A., Malik, S., Banerjee, S., Tran, V., Li, N., Yin, Z., Kurniawan, F., Orellana, K. & Aihara, H. (2018). *Nucleic Acids Res.* **46**, 10474–10488. 10.1093/nar/gky776 PMC6212786 30169742

[bb97] Stelzer, R., Rzoska-Smith, E., Gundesø, S., Rothweiler, U. & Williamson, A. (2024). *J. Vis. Exp.*, e66930. 10.3791/66930 39037258

[bb16] Terwilliger, T. C., Adams, P. D., Read, R. J., McCoy, A. J., Moriarty, N. W., Grosse-Kunstleve, R. W., Afonine, P. V., Zwart, P. H. & Hung, L.-W. (2009). *Acta Cryst.* D**65**, 582–601. 10.1107/S0907444909012098 PMC2685735 19465773

[bb17] Tomkinson, A. E., Vijayakumar, S., Pascal, J. M. & Ellenberger, T. (2006). *Chem. Rev.* **106**, 687–699. 10.1021/cr040498d 16464020

[bb18] Weiss, B. & Richardson, C. C. (1967). *Proc. Natl Acad. Sci. USA*, **57**, 1021–1028. 10.1073/pnas.57.4.1021 PMC224649 5340583

[bb19] Williamson, A., Grgic, M. & Leiros, H.-K. S. (2018). *Nucleic Acids Res.* **46**, 8616–8629. 10.1093/nar/gky622 PMC6144786 30007325

[bb20] Williamson, A., Hjerde, E. & Kahlke, T. (2016). *Mol. Microbiol.* **99**, 274–290. 10.1111/mmi.13229 26412580

[bb21] Williamson, A. & Leiros, H.-K. S. (2019). *Nucleic Acids Res.* **47**, 7147–7162. 10.1093/nar/gkz596 PMC6698739 31312841

[bb22] Williamson, A. & Leiros, H.-K. S. (2020). *Nucleic Acids Res.* **48**, 8225–8242. 10.1093/nar/gkaa307 PMC7470946 32365176

