# Supplementary material for: Crystal structure of ATP-dependent DNA ligase from Rhizobium phage vB_RleM_P10VF
Source: Acta Crystallogr F Struct Biol Commun. 2025 May 14;81(Pt 6):249–54. doi: 10.1107/S2053230X2500411X (PMC12121391; doi:10.1107/S2053230X2500411X)
Supplement: Supplementary file 1 [file f-81-00249-sup1.pdf]

Acta Cryst F

STRUCTURAL BIOLOGY
COMMUNICATIONS

Volume 81 (2025)

Supporting information for article:

Crystal structure of ATP-dependent DNA ligase from *Rhizobium* phage vB_RleM_P10VF

Ulli Rothweiler, Hanna-Kirsti S Leiros and Adele Williamson

Supplementary S1- Expression and purification of native protein for enzymatic assays

The genes encoding DNA ligases from P10VF-Lig from *Rhizobium* phage vB_RleM_P10VF (YP_009099956) and Ac42-Lig from *Acinetobacter* phage Ac42 (YP_004009565) were synthesized by GeneArt (Life Technologies) with codon optimization for expression in *Escherichia coli.* Clonal genes, which also encoded an N-terminal cleavage site for the Tobacco Etch Virus (TEV) protease, were purchased pre-cloned into pDONR221 entry vectors and then subcloned into pDEST17 expression vectors using Gateway recombination.

Standard expression of unlabelled P10VF-Lig and Ac42-Lig for enzyme assays was carried out in *E. coli* strain BL21 pLysS (DE3) by growth on Terrific Broth (TB) media amended with 50 µg/mL carbenicillin at 37 °C with shaking (200rpm) until an OD_{600} of 0.3 was reached. After this time the growth temperature was decreased to 15 °C (P10VF-Lig) or 20 °C (Ac42-Lig) and protein expression was induced by addition of 0.1 mM IPTG (P10VF-Lig) or 0.5 mM IPTG (Ac42-Lig). Cells were harvested by centrifugation at 6000 xg for 10 min, then resuspended in lysis buffer (50 mM Tris pH 8.0, 750 mM NaCl, 1 mM $MgCl_2$, 0.1 mM ATP, 5% glycerol) before lysis by two passes through a French press cell disruptor. Cell harvesting, disruption and all subsequent purification steps were carried out at 4 °C. Cell lysate was clarified by centrifugation at 20,000 xg and the supernatant was applied to a 5 mL immobilised metal affinity chromatography (IMAC) His-Trap column (Cytiva), washed with 20 column volumes of Buffer A (50 mM Tris pH 8.0, 750 mM NaCl, 50 mM imidazole, 5% glycerol) and eluted on a linear gradient from in the same buffer containing 500 mM imidazole. IMAC elution fractions containing P10VF-Lig were pooled, up-concentrated using 30 kDa cutoff centrifugal concentrators (Amicon), exchanged into TEV-cleavage buffer (50 mM Tris pH 8.0, 100 mM NaCl, 5% glycerol 1 mM DTT, 0.5 mM EDTA) using a HiPrep 26/10 desalting column (Cytiva) and incubated with 0.1 mg/mL TEV protease overnight. After incubation, this was re-applied to a 5 mL His-trap pre-equilibrated with TEV cleavage buffer to remove the TEV protease and the residual His-tag. Column flow through which contained the untagged P10VF-Lig was up concentrated again and applied to a HiLoad 16/600 Superdex 200 gel filtration column then eluted in TEV cleavage buffer. Protein-containing fractions from the single gel-filtration peak were concentrated to 2 mg/mL and mixed with 50% v/v glycerol for long-term storage at -80 °C. IMAC elution fractions

containing Ac42-Lig were pooled, up-concentrated and applied directly to the HiLoad 16/600 Superdex 200 gel filtration column without removal of the His-tag. Eluted fractions were concentrated and stored in the same way as P10VF-Lig.

Pmar-LigP and T4 DNA ligase were expressed and purified as described previously (Williamson & Leiros, 2019).

Supplementary S2- DNA ligase assay

DNA ligase activity was measured on substrates containing single nicks or double-stranded breaks as described previously (Stelzer *et al.*, 2024, Williamson & Leiros, 2019, Williamson *et al.*, 2018). Briefly, fluorescently-labelled substrate duplexes were annealed from oligonucleotides and the reaction was assembled to give 80 nM DNA substrate, 1.0 mM ATP, 10 mM $MgCl_2$, 1.0 mM 1,4-Dithiothreitol (DTT), 100 mM NaCl, 50 mM Tris pH 8.0. Blunt and T:A substrate reactions included 10% w/v PEG3350.

DNA ligases were reacted with each substrate under the following conditions: nicked substrate 20 nM ligase incubated for 15 min at 25°C; cohesive overhang substrate 50 nM ligase incubated for 2 h 25°C; blunt substrate and T:A overhang substrate 500 nM ligase incubated for 18 h 15°C. Reaction products were electrophoresed on urea PAGE gels, imaged and analysed as described (Stelzer *et al.*, 2024).

Supplementary figure S3- Sequence alignment of P10VF-Lig with T4 DNA ligase and other homologs

Sequence alignment of P10VF-Lig , Ac42-Lig, Pmar-LigP and T4 DNA ligase. Alignment was done in Geneious Prime 2025.0.3 and visualised using ESPript 3.0 ENDscript - https://endscript.ibcp.fr, (Robert & Gouet, 2014). Secondary structures are shown for P10VF-Lig (top) and T4 DNA ligase (bottom). Partially conserved residues in the alignment are shown in red text; fully conserved positions in white with red background. Structural domain boundaries for the DNA-binding, nucleotidyl transferase and oligonucleotide-binding domains are annotated (gold, red and cyan respectively). Structural elements discussed in the body text which are present in T4 DNA ligase but either absent or unstructured in P10VF-Lig are highlighted in yellow.

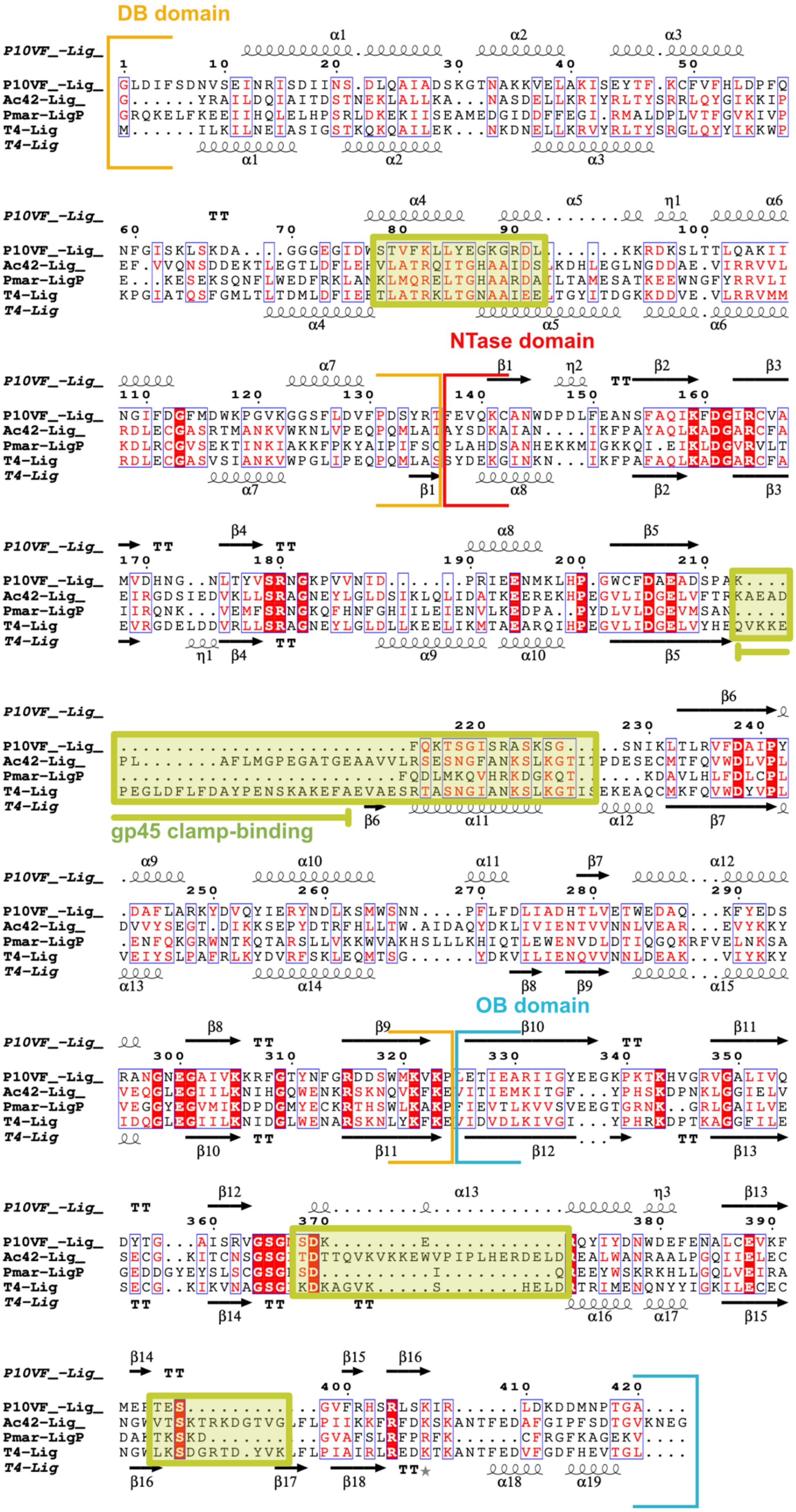
DB domain
NTase domain
OB domain
gp45 clamp-binding
P10VF_-Lig_
Ac42-Lig_
Pmar-LigP
T4-Lig

Supplementary table S4- Sequence identity of P10VF with T4 DNA ligase and other homologs

Sequence identity for aligned DNA ligases P10VF-Lig , Ac42-Lig, Pmar-LigP and T4 DNA ligase. Alignment was done in Geneious Prime 2025.0.3 using the 'global alignment with end gaps' option with the Blosum62 cost matrix, gap open penalty 12, extension penalty 3 and two iterations of refinement.

	**P10VF-Lig**	**Ac42-Lig**	**Pmar-LigP**	**T4-Lig**
**P10VF-Lig**	100%	15.6%	16.6%	17.2%
**Ac42-Lig**		100%	19.9%	52.5%
**Pmar-LigP**			100%	17.5%
**T4-Lig**				100%

Supplementary table S5- Interface analysis of DNA. Ligases with DNA substrates

Interface analysis of DNA ligase structures P10VF-Lig, T4 DNA ligase (6DT1) and, Pmar-LigP (6RCE) was done using PDBePISA (Proteins, Interfaces, Structures and Assemblies) accessed at https://www.ebi.ac.uk/pdbe/pisa/pistart.html (Krissinel & Henrick, 2007).

	**P10VF-Lig**				**T4-Lig**				**Pmar-LigP**			
	**DNA**		**Protein**		**DNA**		**Protein**		**DNA**		**Protein**	
**Number of atoms**												
interface	87	20.20%	101	3.20%	139	32.80%	152	4.20%	141	32.90%	154	4.50%
surface	361	84.00%	1843	59.20%	361	85.10%	2068	56.50%	359	83.70%	2013	58.30%
total	430	100.00%	3114	100.00%	424	100.00%	3660	100.00%	429	100.00%	3453	100.00%
**Number of residues**												
interface	12	57.10%	36	9.30%	18	85.70%	52	11.30%	18	85.70%	50	11.60%
surface	21	100.00%	364	94.30%	21	100.00%	421	91.70%	21	100.00%	405	93.80%
total	21	100.00%	386	100.00%	21	100.00%	459	100.00%	21	100.00%	432	100.00%
**Solvent-accessible area, Å**												
interface	901.7	18.30%	742.4	3.70%	1406.6	29.20%	1206.7	5.10%	1386.6	28.40%	1165.3	5.00%
total	4919.3	100.00%	20107.4	100.00%	4825.1	100.00%	23536.5	100.00%	4879.5	100.00%	23178.4	100.00%
**Solvation energy, kcal/mol**												
isolated structure	41	100.00%	-337.8	100.00%	36.7	100.00%	-421.3	100.00%	44.2	100.00%	-380.8	100.00%
gain on complex formation	-14.3	-34.80%	-1	0.30%	-14.2	-38.70%	-0.1	0.00%	-18.2	-41.20%	-8.8	2.30%
average gain	-10	-24.40%	-1.2	0.40%	-14.3	-38.80%	-3.1	0.70%	-17.6	-39.80%	-3.8	1.00%
P-value	0.27		0.55		0.496		0.761		0.466		0.097	

References

Krissinel, E. & Henrick, K. (2007). *J Mol Biol* **372**, 774-797.

Robert, X. & Gouet, P. (2014). *Nucleic Acids Res* **42**, W320-324.

Stelzer, R., Rzoska-Smith, E., Gundesø, S., Rothweiler, U. & Williamson, A. (2024). *Journal of visualized experiments : JoVE*.

Williamson, A., Grgic, M. & Leiros, H. S. (2018). *Nucleic Acids Res* **46**, 8616-8629.

Williamson, A. & Leiros, H. S. (2019). *Nucleic Acids Res* **47**, 7147-7162.
